# Neural mechanisms for spatial computation

**DOI:** 10.1113/JP273087

**Published:** 2016-11-15

**Authors:** Matthew F. Nolan

**Affiliations:** ^1^ Centre for Integrative PhysiologyUniversity of EdinburghEdinburghEH8 9XDUK

Locating oneself within an environment, remembering goal locations and planning routes are fundamental cognitive functions (Wolbers & Hegarty, 2010). Profound insights into the mechanisms for these cognitive abilities have come from the discovery of neurons in the hippocampus and entorhinal cortex with spatial firing properties. These neurons, which include place cells (O'Keefe, 1976), head direction cells (Taube *et al*. 1990), grid cells (Hafting *et al*. 2005) and border cells, also called boundary cells (Solstad *et al*. 2008; Lever *et al*. 2009), have stereotypical spatial firing patterns, leading to the idea that the hippocampus, entorhinal cortex and their associated structures form a neural system for spatial computation. In part because of the robustness of these spatial codes, the hippocampus and its associated cortical structures have become a major focus for investigation of physiological mechanisms underlying cognitive function (Moser & Moser, 2013).

While firing properties of place, head direction, grid and other spatial cells have been described in considerable detail, the mechanisms responsible for spatial firing and the precise roles in behaviour of these and other less well‐characterized cells remain the subject of considerable investigation and debate. Recently, attention has focused on the computations that hippocampal and entorhinal circuits carry out and the circuit mechanisms that enable these computations to take place. These topics were the focus of a symposium entitled ‘Knowing where you are: circuit mechanisms for estimating location’ and a satellite workshop entitled ‘Spatial computation: from neural circuits to robot navigation’ held at the British Neuroscience Association meeting in Edinburgh in April 2015. Speakers from these symposia have contributed the following symposium reviews for *The Journal of Physiology* related to the work that they presented. The reviews aim to address a number of fundamental questions.

How well do we understand the codes generated by spatial neurons? The striking and robust spatial periodicity of grid firing fields has stimulated the idea that grid cells encode a universal metric for space. Recent experimental results by Krupic and colleagues challenge this idea by showing that grid fields become distorted in asymmetric environments (Krupic *et al*. 2015). In their review, Krupic *et al*. (2016) put these results in the context of investigations of neural representations of geometric cues. They propose that instead of encoding a metric for space, grid cells are part of a neural system for representing geometric features of an environment. Based on this framework, they suggest how environmental boundaries may play an important role in establishing grid firing.

How is the firing of grid cells anchored to an environment? Grid cells are hypothesized to perform path integration; that is, computation of location from information about direction and speed of movement relative to a known starting point. Theoretical analyses indicate that path integration accumulates error with time away from a known location, with the result that grid representations of an environment should drift. By carefully analysing the stability of grid cell patterns in open arenas, Giocomo and colleagues recently found evidence that boundaries anchor grid cell representations (Hardcastle *et al*. 2015). In her review, Giocomo gives an account of the possible roles of environmental boundaries as anchors for the periodic firing fields of grid cells (Giocomo, 2016). She goes on to consider potential involvement of border cells and discusses possible mechanisms for integration of boundary signals with self‐motion signals.

How are grid fields updated during movement? Many models assume that grid fields are generated by integration of direction and speed signals. Although head direction cells at first appear to be a good candidate for the required direction signal, recent work by Hasselmo and colleagues suggests that this is unlikely, as head orientation is often different from movement direction (Raudies *et al*. 2015). They went on to develop models demonstrating how grid firing patterns could be updated either from optic flow or from static visual cues (Raudies *et al*. 2012; Raudies & Hasselmo, 2015). In their review, Hasselmo and colleagues relate these findings to models for grid firing, compare possible roles of visual and other sources of self‐motion information and evaluate mechanisms by which visual signals might influence grid cells (Raudies *et al*. 2016). They suggest that static features on walls could update grid fields with wide spacing, while signals from the ground may influence fields with narrow spacing.

What happens when signals used to anchor spatial firing are unreliable or in conflict? There is evidence that in sensory systems optimal estimates for cue integration are generated based on prior experience in combination with immediate sensory input (e.g. Ernst & Banks, 2002), but it is not clear whether spatial systems operate in a similar fashion. Jeffery *et al*. (2016) consider this problem from the perspective of the head direction system. They review evidence that spatial systems account for cue reliability and then consider how this could be achieved in ring attractor circuits proposed to account for head direction firing. They show how short‐term plasticity of afferent synapses could be used for short‐term cue integration, while longer‐term plasticity might be used to dissociate irrelevant from informative cues.

How are sensory and spatial signals integrated? Estimates of location and heading can be updated either using sensory information, for example from visual landmarks, or using internally generated self‐motion information. Evans *et al*. (2016) discuss the relative roles of sensory and self‐motion signals in generation of place, head direction, boundary and grid firing fields. They describe parallel circuit mechanisms for transformation of sensory information into spatial signals, identify neocortical circuits that may mediate important transformation of egocentric (self‐related) into allocentric (environment‐related) signals and highlight the importance of interactions between different spatial cell types for generation of coherent spatial representations.

How do cellular properties of neurons and their organization account for spatial computations? Recent work suggests that grid firing and associated theta (θ) nested gamma (γ) oscillations can be accounted for by interactions between excitatory and inhibitory cell populations (Pastoll *et al*. 2013). Modulation of these shared interactions may enable independent control of γ oscillations and grid firing share (Solanka *et al*. 2015). Shipston‐Sharman *et al*. (2016) discuss implications of experimentally determined features of excitatory–inhibitory connectivity for hypothesized continuous attractor network models of grid firing. They evaluate successes and limitations of existing models in accounting for experimental observations, identify future experimental tests of the attractor network idea and discuss constraints on circuit‐level implementation.

Finally, can engineering approaches to navigation give insights of relevance to neural systems for computing location? Robots equipped with grid cells are able to solve challenging navigational tasks incorporating sensory uncertainty, demonstrating the potential power of robotic systems for investigating neural mechanisms for spatial cognition (Milford *et al*. 2010). In their review, Milford and colleagues highlight areas in which robotics systems have given insight into corresponding neural mechanisms for perception and motor control (Stratton *et al*. 2016). They suggest how similar strategies can be applied to higher‐level cognitive functions and argue that this is particularly well suited to investigation of mechanisms for navigation. They argue that because of the inherent complexity of the real world and of neural systems, including those used for navigation, implementation on robotic devices may become an important supplement to more conventional simulation‐based approaches to testing biological models for spatial computation.

## Additional information

### Competing interests

None declared.

### Funding

The symposium ‘Knowing where you are: circuit mechanisms for estimating location’ was supported by the British Neuroscience Association. The satellite workshop ‘Spatial computation: from neural circuits to robot navigation’ was supported by *The Journal of Physiology* and by the BBSRC (BB/L010496/1).
